# Impact of Circulating Anti-Spike Protein Antibody Levels on Multi-Organ Long COVID Symptoms

**DOI:** 10.3390/vaccines12060610

**Published:** 2024-06-03

**Authors:** Kevin Hamzaraj, Emilie Han, Ena Hasimbegovic, Laura Poschenreiter, Anja Vavrikova, Dominika Lukovic, Lisbona Kastrati, Jutta Bergler-Klein, Mariann Gyöngyösi

**Affiliations:** Department of Internal Medicine II, Division of Cardiology, Medical University of Vienna, 1090 Vienna, Austria; kevin.hamzaraj@meduniwien.ac.at (K.H.); emilie.han@meduniwien.ac.at (E.H.); ena.hasimbegovic@meduniwien.ac.at (E.H.); laura.poschenreiter@meduniwien.ac.at (L.P.); anja.vavrikova@meduniwien.ac.at (A.V.); dominika.lukovic@meduniwien.ac.at (D.L.); lisbona.kastrati@lilienfeld.lknoe.at (L.K.); jutta.bergler-klein@meduniwien.ac.at (J.B.-K.)

**Keywords:** long COVID, vaccine, anti-spike protein antibody, multiorgan symptoms, COVID-19, post-COVID syndrome

## Abstract

Patients with long COVID syndrome present with various symptoms affecting multiple organs. Vaccination before or after SARS-CoV-2 infection appears to reduce the incidence of long COVID or at least limit symptom deterioration. However, the impact of vaccination on the severity and extent of multi-organ long COVID symptoms and the relationship between the circulating anti-spike protein antibody levels and the severity and extent of multi-organ symptoms are unclear. This prospective cohort study included 198 patients with previous PCR-verified SARS-CoV-2 infection who met the criteria for long COVID syndrome. Patients were divided into vaccinated (*n* = 138, 69.7%) or unvaccinated (*n* = 60, 30.3%) groups. Anti-spike protein antibody levels were determined at initial clinical presentation and compared between the groups. Long COVID symptoms were quantified on the basis of the number of affected organs: Class I (mild) with symptoms in three organs, Class II (moderate) with symptoms in four to five organs, and Class III (severe) with symptoms in six or more organ systems. Associations between time to infection and vaccination with anti-spike protein antibody levels were assessed. The anti-spike protein antibody levels were 1925 ± 938 vs. 481 ± 768 BAU/mL (*p* < 0.001) in the vaccinated vs. unvaccinated patients. The circulating anti-spike antibody cutoff of 665.5 BAU/mL allowed us to differentiate the vaccinated from the unvaccinated patients. Vaccinated patients had fewer class II and class III multi-organ symptoms (Class II 39.9% vs. 45.0%; Class III 10.1% vs. 23.3%, *p*-value 0.014). Anti-spike antibody level correlated negatively with multi-organ symptom classes (*p* = 0.016; 95% CI −1.229 to −0.126). Anti-spike antibody levels in unvaccinated patients declined markedly with time, in contrast to the persistence of high anti-spike antibody levels in the vaccinated patients. Multi-organ symptoms were lower in vaccinated long-COVID patients, especially in those with higher anti-spike antibody levels (≥665.5 BAU/mL). Classifying the symptoms on the basis of the number of affected organs enables a more objective symptom quantification.

## 1. Introduction

The COVID-19 pandemic has left a significant proportion of individuals, i.e., over 5% of the total infected population and up to 37% in specific populations, with symptom sequelae following SARS-CoV-2 infection [1,2]. The concept of post-COVID-19 or long COVID syndrome emerged in May 2020 with initial reports of continued or developing symptoms beyond 12 weeks following acute SARS-CoV-2 infection [3,4]. Individuals with long COVID present with symptoms involving multiple organ systems, often manifesting as a cluster of symptoms, with fatigue, headache, and dyspnea being commonly reported [5,6]. Furthermore, cognitive and cardiovascular symptoms, along with bodily pain, are frequent long COVID-associated problems, some of which may be reversed while others persist for at least 12 months post infection [1].

Since the introduction of vaccination against SARS-CoV-2 in late 2020, speculation has arisen regarding a potential vaccine-induced modulation of long COVID disease persistence, influencing the extent of multiple organ involvement and symptom severity. While some studies have failed to demonstrate a correlation between vaccination and long COVID risk, recent data have indeed supported a protective effect of the vaccine in the context of long COVID [7,8]. The administration of a higher number of vaccine doses prior to infection has also been shown to effectively reduce long COVID prevalence as compared with no vaccination at all. [9] This reduction may be attributed to a milder infection during the acute phase due to a protective effect of vaccination, and subsequently, lower long COVID prevalence. Some studies have explored the timing of vaccination relative to infection, with some indicating a partial protective effect of vaccination in patients experiencing breakthrough infections [10]. Moreover, vaccination post infection does not exacerbate long COVID symptoms, which also suggests a protective effect [11].

In fact, data regarding the infection- or vaccine-induced humoral response against SARS-CoV-2 in long COVID cohorts are scarce. However, available data primarily suggest symptomatic improvement with higher antibody levels due to vaccination; there are more robust data on the levels of anti-nucleocapsid protein antibodies [12,13]. Another prospective study revealed no correlation between vaccination and symptom improvement in post-acute COVID-19 syndrome [14]. Consequently, there is a need to investigate the effect of the long-term presence of specific antibodies in cohorts of patients with long COVID, particularly in the absence of dedicated treatments. For this reason, we aimed to analyze quantitative data on circulating anti-spike protein antibody levels in connection with multi-organ symptom extent in a multi-organ assessment of the scale and effect of vaccination within prospectively enrolled patients with long COVID.

## 2. Methods

### 2.1. Study Population

Consecutive patients with the long COVID syndrome presenting to our dedicated outpatient clinic were enrolled as part of a single-center prospective registry (the POSTCOV registry, EC Number: 1008/2021 and 1758/2022; ClinicalTrials.gov identifier: NCT05398952). Patients provided written informed consent for data collection and blood sampling during consecutive visits as part of outpatient clinical care. This registry included patients with prior quantitative real-time PCR-confirmed SARS-CoV-2 infection who had experienced mild to moderate COVID-19, i.e., not requiring hospitalization, and no history or current inflammatory diseases, chronic organ disorders, or malignancies. All patients exhibited symptoms originating from at least three organs, meeting the criteria for long COVID syndrome [5]. Patients with elevated inflammatory markers were excluded from the study. Furthermore, patients with symptoms suggestive of vaccine-induced long COVID but lacking prior confirmed SARS-CoV-2 infection were also excluded from the study. Each visit occurred within 48 h of a negative SARS-CoV-2 quantitative real-time PCR test result.

### 2.2. Clinical and Laboratory Data

Baseline clinical data collection was conducted between April 2021 and May 2022 in a systematic, predefined manner. Collected data included clinical investigations, such as anamnesis, comorbidities, date of prior infections, and date of vaccinations.

Symptoms were collected through unconstrained free-form interviews with patients and were categorized according to their primary association with organs: neurological, pulmonary, cardiac, olfactory and gustatory, gastrointestinal, dermatological, musculoskeletal and metabolic, or hematological organ-related symptoms. Patients were classified into symptom clusters on the basis of the number of involved organs: Class I (mild) with symptoms in three organs, Class II (moderate) with symptoms in four to five organs, and Class III (severe) with symptoms in six or more organs. The clustering method was based on our previous observations, the literature, and the specifications outlined in the international Delphi consensus [1,3,6,15,16,17]. Our classification notably aligns with the definitions provided by Ayoubkhani et al., who quantified individual symptoms in a large prospective out-of-hospital study and categorized them into three groups, similar to ours, on the basis of quantitative observations [11].

Blood sampling was performed at the baseline clinical presentation. Routine laboratory tests including hematology, clinical chemistry tests, inflammation, and autoimmune marker tests were performed at the Department of Laboratory Medicine, Medical University of Vienna, Vienna, Austria. All laboratory parameters, including the anti-spike protein antibody quantification, were measured according to protocols outlined on the homepage of the Department of Laboratory Medicine (https://www.akhwien.at/default.aspx?pid=3985, accessed on 26 April 2024).

Anti-spike protein antibodies were quantified from serum samples previously stored in our biobank repository at −80 °C. Analyses were performed in a Cobas e 801 unit (Roche Diagnostics, Rotkreuz, Switzerland) employing the Elecsys Anti-SARS-CoV-2 S spike protein electro-chemiluminescence immunoassay (Roche Diagnostics) for in vitro quantitative measurement of antibodies to the SARS-CoV-2 spike protein, following the manufacturer’s instructions. This assay utilizes a recombinant receptor-binding domain protein as a double-antigen sandwich assay and favors high-affinity antibodies.

### 2.3. Hypothesis and Aims

We hypothesized that vaccinated patients with long COVID would exhibit higher circulating SARS-CoV-2 anti-spike protein antibody levels associated with lower multi-organ symptom severity and extent than would unvaccinated individuals.

### 2.4. Statistical Analyses

Descriptive statistics of demographic data and clinical parameters were obtained by categorizing the cohort into vaccinated and unvaccinated patients and expressing variables as frequencies and percentages or as means and standard deviations. For statistical significance, the chi-square test, Fisher exact test, Student *t*-test, or Mann-Whitney U test was employed. Statistical significance was indicated by a two-sided *p*-value of <0.05.

The anti-spike protein antibody levels were compared between vaccinated and unvaccinated patients with long COVID using the two-sided Mann-Whitney U test. Benjamini-Hochberg correction for multiple testing was applied for clinical and vaccination- and infection-related parameters, and adjusted levels of significance were used to confirm the significance of variables of interest. A receiver operating characteristic (ROC) curve analysis was conducted to determine an optimum cutoff value of anti-spike protein antibody level to distinguish between vaccinated and unvaccinated patients with long-COVID. This cutoff value was validated using binary logistic regression.

The correlation between vaccination status at blood sampling and symptom extent was tested using ordinal regression, with symptom extent expressed both quantitatively and on the three-class symptom scale. The regression analysis was repeated using the circulating anti-spike protein antibody cutoff in order to validate its association with symptom extent, similar to vaccination status.

A cubic curve estimation of circulating anti-spike protein antibody levels was conducted relative to time from infection or first vaccine to blood sampling and from infection to vaccination.

## 3. Results

A total of 198 patients on the POSTCOV prospective registry with quantitative anti-spike protein antibody titer were included in the study. Detailed demographics are presented at the Table 1. The cohort consisted predominantly of female patients and mostly patients at a younger age. The most prevalent comorbidities at the time of enrollment included hypertension and hyperlipidemia, while the largest proportion of the cohort had no prior disease or medical conditions. Patients exhibited symptoms mostly affecting three organs (Class I), followed by four to five organs (Class II), with only a small proportion experiencing symptoms affecting ≥6 organs (Class III). The mean time from the first confirmed SARS-CoV-2 infection to the initial presentation at our center after symptom onset or continuity was 246 ± 152 days (8 ± 4 months).

### 3.1. Vaccination Status and Long COVID Symptoms

Among our long COVID patient cohort, 138 patients had received at least one vaccine dose at the time of first presentation, while 60 patients had not yet received any vaccine dose. All vaccinated patients had received monovalent mRNA or adenoviral vector vaccines against SARS-CoV-2, i.e., one of the four commercial products available in Austria at the time, developed/marketed by the companies Pfizer (New York, NY, USA)—BioNTech (Mainz, Germany), Oxford-AstraZeneca (Cambridge, UK), Janssen (Beerse, Belgium), Moderna (Cambridge, MA, USA). At the first presentation, 83.3% of patients had been vaccinated against SARS-CoV-2 after an infection, while the remaining patients had breakthrough infections after receiving at least the first vaccine dose. Most patients received the Pfizer-BioNTech Comirnaty vaccine at first, with all patients that had been boosted receiving only this highly available mRNA-based vaccine.

**Figure 1:**
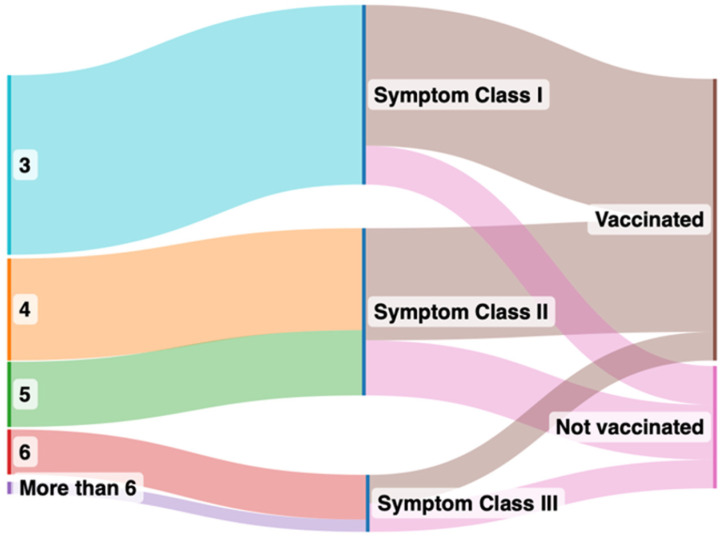
Sankey plot comparing symptoms after grouping into vaccinated and unvaccinated groups. The left panel indicates number of organs affected by long COVID symptoms after clustering, the right panel indicates the symptom classes from I to III in vaccinated (brown color in the right panel) and unvaccinated (pink in the right panel) groups.

Vaccinated and unvaccinated patients at the time of first presentation had similar past medical histories, vital parameters, and laboratory values (Table 1). However, vaccinated patients had notably higher circulating SARS-CoV-2 anti-spike protein antibody levels and experienced fewer long COVID symptoms, belonging to Class I, as compared to unvaccinated patients (Figure 1). Class II symptoms were the most prevalent in unvaccinated patients and significantly more frequent as compared with the vaccinated patients. While Class III symptoms were least prevalent in both groups, they were significantly more prevalent in the unvaccinated patients. The unvaccinated patients came to report their symptoms earlier relative to their first infection (6 ± 4 months) compared with their vaccinated counterparts (9 ± 5 months).

### 3.2. Time-Dependent Decrease in Levels of Circulating SARS-CoV-2 Anti-Spike Protein Antibodies in the Two Groups

The study cohort generally had considerable levels of circulating SARS-CoV-2 anti-spike protein antibodies, with vaccinated patients having significantly higher levels than unvaccinated patients (Table 1).

**Figure 2:**
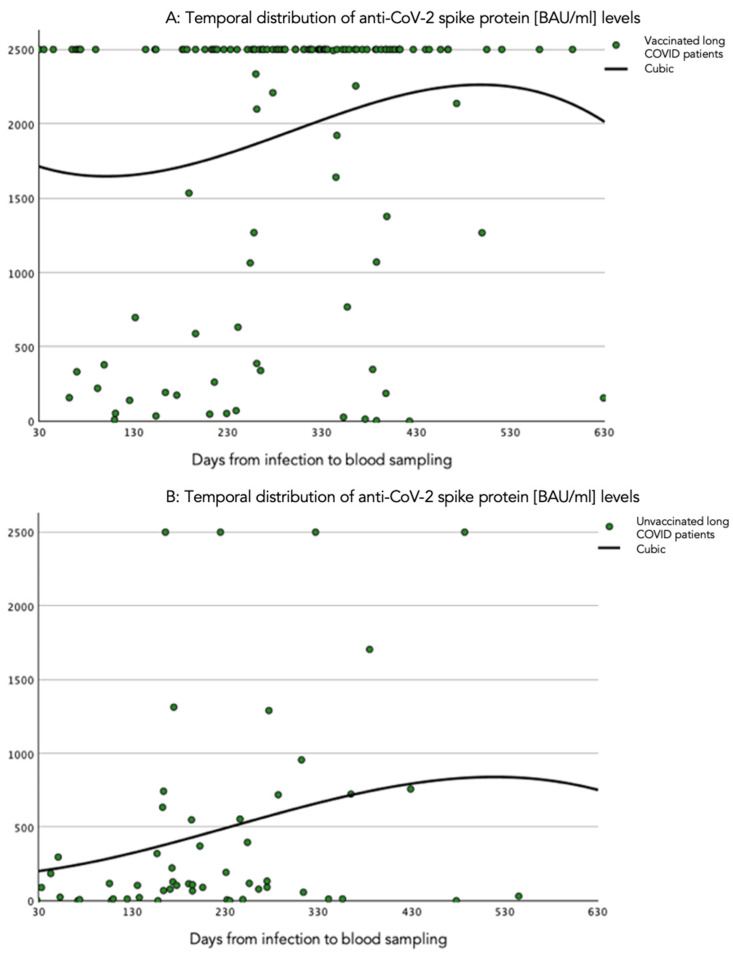
Cubic spline representation of the relation of quantitative circulating SARS-CoV-2 anti-spike protein antibody levels and time windows. (**A**) Time period (days) from infection to blood sampling in vaccinated patients with long COVID. (**B**) Time period (days) from infection to blood sampling in unvaccinated patients with long COVID. The anti-SARS-CoV-2 spike protein antibody levels are presented in BAU/mL.

The temporal course of the anti-spike protein antibody levels was investigated in various time windows using cubic spline estimation, as presented in Figure 2. The anti-spike protein antibody level tended to increase after infection, influenced by values over the detection limit. In contrast, anti-spike protein antibody levels remained low and decreased over time in unvaccinated patients.

### 3.3. Determination of a Cutoff Value for Anti-Spike Protein Antibody Characterizing Vaccinated versus Unvaccinated Patients

The Mann-Whitney U test revealed statistically significant differences between the two groups regarding anti-spike protein antibody levels, and a correlation was established by logistic regression (*p*-value < 0.001). Our ordinal regression analysis indicated a correlation of anti-spike protein antibody levels and symptom class (*p*-value = 0.017), with a good fit, as tested by goodness of fit and a test of parallel lines.

**Figure 3:**
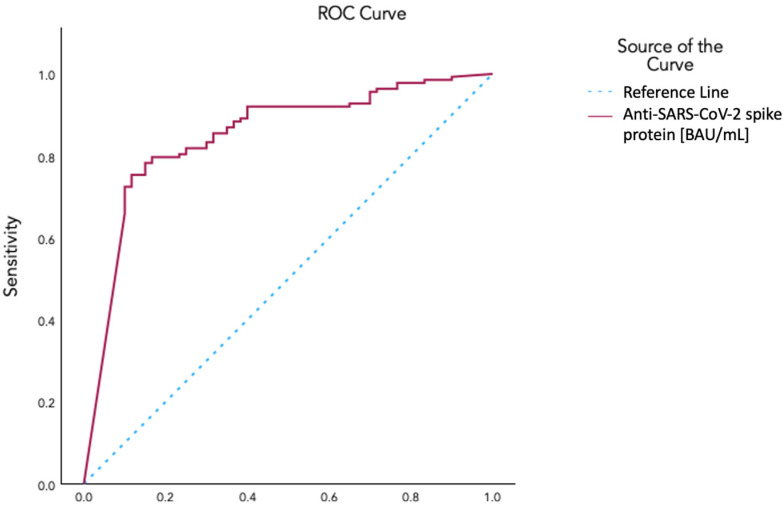
ROC curve for the determination of the cutoff value of circulating SARS-CoV-2 anti-spike protein antibody levels for characterization of vaccination status using AUC: 0.848; Cutoff Coordinate: 665.5 BAU/mL in a Sensitivity 0.804 and 1−Specificity 0.233; Youden’s Index 0.571.

An ROC curve analysis yielded an area under the curve of 0.848 for identifying vaccinated as opposed to unvaccinated patients on the basis of anti-spike protein antibody levels. A value of 665.5 BAU/mL was selected with a sensitivity of 0.804, a 1-specificity of 0.233, and a Yoden’s index of 0.571 (Figure 3). Using this value as a cutoff, we determined a threshold anti-spike protein antibody level for vaccinated vs. unvaccinated conditions. Aiming to explore the correlation with symptom class after dichotomizing patients with this threshold, we used ordinal regression, which revealed a negative correlation with symptom classes (*p*-value 0.016; 95% CI −1.229 to −0.126).

### 3.4. Subgroup Analysis of Patients with Low versus High Anti-Spike Protein Antibody Levels

Table 2 visualizes the group statistics after dichotomizing the patients by anti-spike protein antibody levels into high or low subgroups. In the low anti-spike group (<665.5 BAU/mL), 27 patients (37%) were vaccinated, and 50 patients (83.3%) were unvaccinated. The Pfizer-BioNTech vaccine was the most frequent in both groups. Patients with high anti-spike protein antibody levels had mostly received two vaccine doses and had a significantly higher mean anti-spike level. Baseline clinical and laboratory parameters did not differ between the groups.

## 4. Discussion

In our study, we identified a cutoff value of 665.5 BAU/mL as an anti-spike protein antibody level that stratified patients by vaccination status and was strongly correlated with multi-organ long COVID symptom extent. Vaccinated patients with long COVID consistently exhibited significantly higher and sustained levels of plasma anti-spike protein antibodies, along with symptoms arising in fewer organs.

Long COVID syndrome has been a topic of discussion for several years, with symptomatic patients continuing to experience effects long after their active infection with SARS-CoV-2 [1]. As this syndrome presents with heterogeneous clinical manifestations, investigators have struggled to standardize reports, and quantifying symptom extent has proved challenging. A systematic review managed to identify and categorize up to 200 long COVID-related symptoms described in the literature [16]. Given the multi-organ nature of long COVID syndrome, systematically categorizing symptoms on the basis of organ involvement can help clinicians to better understand the course, severity, and prognosis of long COVID [18]. In this study, we classified the symptoms using the number of organs they affected. This enabled us to provide a more objective and easy communicable classification system than merely reporting individual symptoms. This system stratified the patients into three groups, potentially simplifying our understanding of the syndrome and communication with patients about their symptoms.

It has also been debated whether vaccination against SARS-CoV-2 influences the course or the symptoms of long COVID [7,8,9,11,19]. Our findings indicate that the multi-organ symptoms were milder in the patients with long COVID who were vaccinated. This aligns with a large meta-analysis suggesting that vaccination before an infection offers an additional protective effect in long COVID symptom severity, with no worsening of symptoms whatsoever in patients vaccinated after infection [8]. Al-Aly et al. compared patients with a breakthrough infection with or without vaccination and historical controls. They showed a lower extent of symptoms after breakthrough infections in vaccinated patients [10]. Another systematic review suggested positive effects of vaccination by stating that, in most studies, long COVID symptoms improved after at least one dose of vaccine. [20] Our study corroborates these findings, providing further insights into multi-organ symptom extent using an unprecedented, simple, and usable system.

Although the etiology of long COVID is still unknown, hypotheses include an interplay of several factors such as systemic immunologic dysfunction or dysautonomia, viral or immune-mediated organ injury, and physical deconditioning [4,21,22]. In this context, determination of anti-spike protein antibody levels could help elucidate the relationship of precedent humoral response to long COVID symptoms. Reports have already speculated about several mechanisms by which the vaccine could influence long COVID incidence, course, and symptoms by activating or modulating the immune system across several organs. While the direction of these changes may vary, observational studies have more commonly articulated a protective effect of the vaccine rather than a deteriorating one [3,5,8,23]. This may result from an immune system modulation or “reset” by the vaccine after the infection, or protection from severe active COVID-19 disease, which reflects lower long COVID incidence or severity.

A few studies have investigated SARS-CoV-2 spike and nucleocapsid antibodies, mainly focusing on their relation to the incidence of post-acute COVID sequelae or fatigue in long COVID [14,24,25,26]. To the best of our knowledge, none has explored binding antibodies in the context of multi-organ symptom severity or extent. In an early study, Blomberg et al. reported a correlation between anti-spike protein antibody levels measured 2 months post infection and long-term fatigue severity in a mixed cohort, including patients with post-COVID sequelae [27]. Wynberg et al. found no difference in binding or neutralizing anti-spike antibodies between matched patients with or without post-acute sequelae, measured shortly after COVID-19. On the other hand, Molnar et al. investigated a long COVID-only cohort and found higher anti-spike and anti-nucleocapsid titers in patients with non-severe fatigue, with a decrease in anti-nucleocapsid antibody levels in severe fatigue patients over time [26]. The same group subsequently reported that only high levels of anti-nucleocapsid antibodies predicted remission from severe fatigue [13]. In our cohort, which exhibited a variable number of vaccinations and different vaccination timelines relative to infection, we opted to focus on anti-spike protein antibody levels over anti-nucleocapsid antibody levels. This decision was based on the predominant mechanism of most SARS-CoV-2 vaccines, which primarily introduce or stimulate the production of and response against the spike glycoprotein. Additionally, we consider that exploring the relationship between binding antibodies and multi-organ symptoms is valuable, given the complexity of long COVID and the involvement of multiple organs.

Our study revealed a correlation between anti-spike protein antibody levels and the extent of multi-organ symptoms, similar to the correlation observed with vaccination status. Patients with higher anti-spike protein antibody levels experienced symptoms arising in fewer organs. While Varnai et al. reported a similar correlation between higher anti-spike and anti-nucleocapsid antibody values and less severe fatigue in patients with long COVID, our study offers a broader and more comprehensive outcome. Our study further implies that by using quantitative data based on anti-spike protein antibody binding, we may easily stratify patients with heterogeneous vaccination status, vaccine doses, infection counts, and timeframes between vaccination and infection. This could serve as a tool to guide further patient management and vaccination recommendations and standardize further clinical studies on the syndrome. In this manner, we could offer adequate medical support for the multi-organ symptoms that significantly impact quality of life.

It is important to interpret these findings cautiously, as they are hypothetical, yet they offer valuable insights into individual patient presentations and needs. An anti-spike protein antibody level below 665.5 BAU/mL could indicate a higher extent of multi-organ symptoms and could complement symptom classification when depicting the trajectory of the syndrome over months of clinical care. Currently, COVID-19 appears to be transitioning into a seasonal disease with occasional severe courses in recent infections among vulnerable populations, followed by post-acute sequelae. There is a need for an attention shift and the use of information from studies like ours in prospective cases to provide better medical assistance in this patient population, which is growing and evolving, even in the post-pandemic era.

### Limitations

Our study was limited by a relatively small sample number and its observational nature. This prospective registry mainly focused on symptoms of long COVID. Furthermore, we only determined the anti-spike protein antibody levels in the first patient presentation and followed up the patients in intervals only by gathering symptom information. Tracking the temporal course of anti-spike protein antibody levels or spontaneous symptom trajectory was not undertaken this study. We limited our analysis to the anti-spike protein antibody and did not measure anti-nucleocapsid antibodies. The majority of infections occurred during the fourth surge of the COVID-19 pandemic, with the Delta variant being predominant. The virus variant carries crucial information about the cross-correlation of binding and neutralizing titers, as the Delta variant resulted in stronger neutralizing titers (WT Wuhan strain) than did the later variants such as Omicron. We did not test for the virus variant, since patients visited the outpatient ward at least 3 months after experiencing acute infection (the definition of long COVID syndrome), and in accordance with the outpatient ward rules during the pandemics, patients had to present a negative PCR test result to enter the hospital for the visit. The vast majority of the patients had an active infection during the “Delta surge”, according to the required document for the inclusion in the registry (PCR-verified SARS-CoV-2 infection).

Here, we aimed to analyze the anti-spike protein antibody level as a marker of humoral response to SARS-CoV-2 infection in unvaccinated and vaccinated patients with long COVID. While the level of immune protection against breakthrough SARS-CoV-2 infection could only be determined by viral neutralization assays and not antibody titers, our study focused solely on investigating multi-organ symptom extent rather than interpreting immunity status or antibody efficacy.

## 5. Conclusions

In conclusion, vaccination appears to protect from greater multi-organ symptom extent in patients with long COVID. A cutoff level of anti-SARS-CoV-2 spike protein antibodies of 665.5 BAU/mL identified patients as vaccinated or unvaccinated and was associated with multi-organ symptom extent.

## Figures and Tables

**Table 1:** Baseline characteristics of vaccinated and unvaccinated patients with long COVID.

Variable	Total *n* = 198	Vaccinated *n* = 138	Unvaccinated *n* = 60	*p*-Value
Age (years)	45.1 ± 14.2	45.5 ± 14.7	44.3 ± 13.2	0.603
Female (%)	139 (70.2)	96 (69.6)	43 (71.7)	0.766
BMI (kg/m^2^)	25.3 ± 5.2	24.9 ± 4.9	26.2 ± 5.8	0.165
Hypertension (%)	73 (36.9)	53 (38.4)	20 (33.3)	0.497
Diabetes mellitus (%)	8 (4.0)	7 (5.1)	2 (1.7)	0.263
Hyperlipidemia (%)	55 (27.8)	38 (27.5)	17 (28.3)	0.908
Family history for CVD (%)	29 (14.6)	21 (15.2)	8 (13.3)	0.730
Symptom Class (%)				**0.014**
I	88 (44.4)	69 (50.0)	19 (31.7)	
II	82 (41.4)	55 (39.9)	27 (45.0)	
III	28 (14.1)	14 (10.1)	14 (23.3)	
Anti-SARS-CoV2-spike (BAU/mL)	1487 ± 1109	1925 ± 938	481 ± 768	**<0.001**
Days from infection to blood sampling (d)	246 ± 152	269 ± 151	193 ± 140	**0.001**
Days from infection to vaccination (d)		110 ± 167		
Vaccine prior to infection (%)	23 (11.6)	23 (16.7)	0 (0)	**-**
Vaccine doses (%)				**<0.001**
0	60 (30.3)	0 (0.0)	60 (100)	
1	43 (21.7)	43 (31.2)		
2	57 (28.8)	57 (41.3)		
3	34 (17.2)	34 (24.6)		
4	4 (2.0)	4 (2.9)		
First vaccine type (%)				-
AstraZeneca	20 (10.1)	20 (14.5)		
Janssen	2 (1.0)	2 (1.4)		
Moderna	12 (6.1)	12 (8.7)		
Pfizer-BioNTech	104 (52.5)	104 (74.4)		
NT-proBNP (mg/dL)	89.1 ± 164	94.8 ± 184.5	76.4 ± 104.4	0.425
CRP (µg/mL)	0.2 ± 0.3	0.2 ± 0.32	0.21 ± 0.28	0.668
TSH (mIU/L)	1.52 ± 0.81	1.53 ± 0.83	1.49 ± 0.78	0.812
IgG (mg/dL)	1126 ± 300	1104 ± 239	1177 ± 404	0.115
Cholesterol (mg/dL)	199 ± 42	197 ± 42	202 ± 41	0.527
Triglycerides (mg/dL)	115.65 ± 78.15	108 ± 71	134 ± 91	0.122
Lp(a) (mg/dL)	49 ± 75	55 ± 82	36 ± 53	0.346
CK (U/L)	99 ± 58	96 ± 51	106 ± 72	0.801
Procalcitonin (ng/mL)	0.03 ± 0.03	0.03 ± 0.02	0.03 ± 0.03	0.930
Ferritin (ng/mL)	118 ± 122	122 ± 125	111 ± 116	0.250
D-Dimer (mg/L FEU)	0.27 ± 0.38	0.31 ± 0.42	0.18 ± 0.21	0.086
IgA (mg/dL)	207 ± 100	204 ± 101	217 ± 97	0.348
Fibrinogen (mg/dL)	319 ± 67	320 ± 69	318 ± 63	0.964
aPTT (s)	35.3 ± 3.6	35.4 ± 3.7	35.1 ± 3.5	0.947
Thrombocytes (/nL)	261 ± 54	260 ± 56	262 ± 50	0.816
Hemoglobin (g/dL)	14.35 ± 2.36	14.4 ± 2.55	14.24 ± 1.92	0.945

BMI—body mass index, CVD—cardiovascular disease, CK—creatinine kinase, aPTT—activated partial thromboplastin clotting time, CRP—C reactive protein, TSH—thyroid-stimulating hormone, Ig—immunoglobulin.

**Table 2:** Patients with low (<665.5) and high (≥665.5) anti-spike levels, as determined by the cutoff value of circulating anti-spike protein antibody.

Variable	Low Anti-Spike Level (<665.5 BAU/mL) *n* = 73	High Anti-Spike Level (≥665.5 BAU/mL) *n* = 125	*p*-Value
Vaccinated (%)	27 (37.0)	111 (88.8)	<0.001
Female (%)	53 (72.6)	86 (68.8)	0.572
Hypertension (%)	26 (35.6)	47 (37.6)	0.780
Diabetes mellitus (%)	1 (1.4)	7 (5.6)	0.145
Hyperlipidemia (%)	18 (24.7)	37 (29.6)	0.454
Family history for CVD (%)	8 (11.0)	21 (16.8)	0.262
Symptom Class (%)			**0.042**
I	24 (32.9)	64 (51.2)	
II	36 (49.3)	46 (36.8)	
III	13 (17.8)	15 (12.0)	
Vaccination (%)	0	0	**<0.001**
before COVID-19	3 (4.1)	20 (16.0)	
after COVID-19	24 (32.9)	91 (72.8)	
Vaccine doses (%)			**<0.001**
0	46 (63.0)	14 (11.2)	
1	11 (15.1)	32 (25.6)	
2	11 (15.2)	46 (36.8)	
3	5 (6.8)	29 (23.2)	
4	0 (0.0)	4 (3.2)	
First vaccine type (%)	4 (5.5)	16 (12.8)	**-**
Astra Zeneca	0 (0)	2 (1.6)	
Janssen	1 (1.4)	11 (8.8)	
Moderna	22 (30.1)	82 (65.6)	
Pfizer-BioNTech	46 (63.0)	14 (11.2)	
SARS-CoV-2 anti-spike (BAU/mL)	146 ± 166	2270 ± 514	**<0.001**
Age (y)	44.0 ± 13.8	45.8 ± 14.4	0.399
BMI (kg/m^2^)	24.9 ± 5.03	25.5 ± 5.24	0.496
Days from infection (d)	189 ± 140	280 ± 149	**<0.001**
NT-proBNP (mg/dL)	73 ± 96.3	98.8 ± 193.3	0.198
CRP (µg/mL)	0.22 ± 0.31	0.19 ± 0.3	0.573
TSH (mIU/L)	1.47 ± 0.71	1.54±0.87	0.555
IgG (mg/dL)	1116 ± 232	1132 ± 334	0.712
Cholesterol (mg/dL)	201 ± 42	197 ± 42	0.445
Triglycerides (mg/dL)	117 ± 76	115 ± 80	0.669
Lp(a) (mg/dL)	36.1 ± 56.2	56.7 ± 84.0	0.448
CK (U/L)	96.6 ± 67.9	100.7 ± 51.2	0.063
Procalcitonin (ng/mL)	0.03 ± 0.03	0.03 ± 0.02	0.774
Ferritin (ng/mL)	145 ±164	102 ± 86	0.236
D-Dimer (mg/L FEU)	0.24±0.29	0.29±0.41	0.796
IgA (mg/dL)	209 ± 90	207 ± 106	0.685
Fibrinogen (mg/dL)	318.4 ± 65.2	319.5 ± 68,4	0.982
aPTT (s)	35.1 ± 3.8	35.4 ± 3.5	0.754
Thrombocytes (/nL)	258 ± 52	261 ± 55	0.674
Hemoglobin (g/dL)	14.19 ± 1.79	14.44 ± 2.65	0.737

BMI—body mass index, CVD—cardiovascular disease, CK—creatinine kinase, aPTT—activated partial thromboplastin clotting time, CRP—C reactive protein, TSH—thyroid-stimulating hormone, Ig—immunoglobulin.

## Data Availability

The data can be shared by the principal investigator up on reasonable request.
